# Early Days in the Hunt Laboratory at UVA, 1969 to 1980

**DOI:** 10.1016/j.mcpro.2024.100874

**Published:** 2024-11-05

**Authors:** P. Jane Gale, George C. Stafford, Howard R. Morris, Charles N. McEwen

**Affiliations:** 1 Gale-Bentz Consulting, Southborough, Massachusetts, USA; 2 Finnigan Corporation/ThermoFisher Scientific, San Jose, CA, USA; 3 Agilent Technologies, Santa Clara, CA, USA; 4 Imperial College, London, UK; 5 Saint Joseph's University, Philadelphia, Pennsylvania, USA

**Keywords:** mass spectrometer, gas chromatograph, liquid chromatograph, chemical ionization, Pulsed Positive Negative Ion Chemical Ionization, Triple Stage Quadrupole

## Abstract

Arriving at the University of Virginia in the autumn of 1969, Donald Hunt began his 50+ year career in academics with the study of organometallic chemistry, on which he had done his PhD thesis work, and mass spectrometry, to which he was introduced while a postdoc in Klaus Biemann’s laboratory at the Massachusetts Institute of Technology. In the 1970s, Hunt’s lab pioneered the use of negative chemical ionization (CI) to enhance sensitivity for studying organic molecules, developed a system for simultaneously obtaining positive and negative CI spectra to augment structure elucidation, and built a prototype triple quadrupole instrument so effective at collisional dissociation that its commercial counterpart became the analytical instrument of choice for mixture analysis for the next decade and beyond. Foreseeing that the future lay in the analysis of biological molecules, by the end of the decade Hunt shifted his focus to peptides. The analysis of protein fragments had suddenly become more accessible thanks to the advent of the triple quadrupole and Barber’s introduction of fast atom bombardment. As the 1980s began and Hunt and his team sought to pursue larger and larger pieces of proteins, his attention turned to the development of mass spectrometers with greater mass range. While recounting their memories of these events, several of Hunt’s students and colleagues pay tribute to his support for them as individuals, as well as to his infectious enthusiasm for scientific endeavors that he so generously shared.

## Highlights

• 1969 Don Hunt joins UVA Chem; research on organometallics and chemical ionization.
• Grad student Stafford develops pulsed pos/neg ion chem ioniz (PPNICI) mass spec.
• Hunt students/postdocs use PPNICI to target EPA priority pollutants in mixtures.
• Grad student Shabanowitz builds prototype triple quad MS after Enke/Yost ASMS talk.
• Hunt convinces Finnigan to commercialize the instrument based on Shabanowitz design.
• With Hunt methodologies, triple quad instruments become choice for mixture analysis.
• After sabbatical, Hunt refocuses lab on peptide analysis with permethylation/FAB.

## Graphical Abstract

**Figure:**
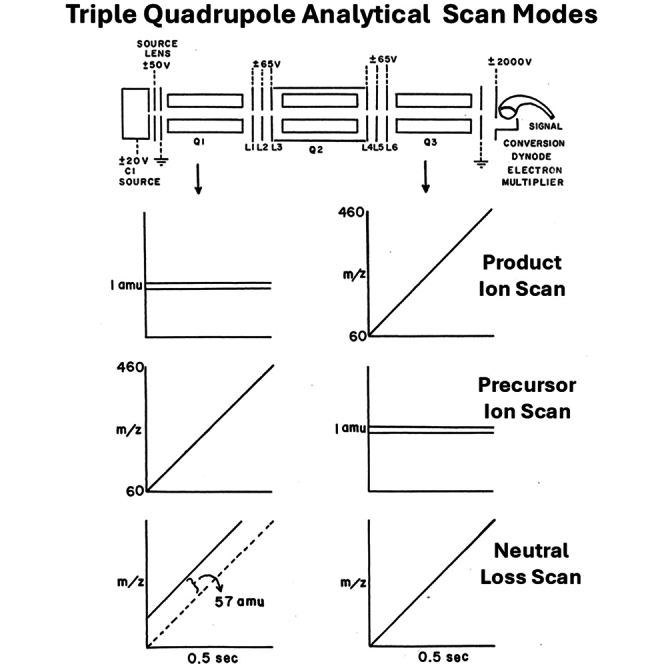

## In Brief

While recounting their memories of the 1970 scientific events in the laboratory of Donald F Hunt at the University of Virginia, several Hunt students and colleagues pay tribute to Hunt’s support for them as individuals, as well as to his infectious enthusiasm for scientific endeavors that he so generously shared.

Don Hunt arrived at the University of Virginia (UVA) as an assistant professor of chemistry in the autumn of 1969. He was fresh out of Klaus Biemann’s lab at the Massachusetts Institute of Technology (MIT) where he had spent a year as a post-doctoral fellow following completion of his PhD at the University of Massachusetts (UMass), Amherst. Continuing to pursue interests he had developed at UMass and MIT (1, 2), he began research programs at UVA in two areas: organometallics and mass spectrometry.

Don’s approach to academia, much like his approach to life we students soon learned, was extremely ordered. There was always a 1-year plan, a 3-year plan, and a 5-year plan on which he was working. His personal notebooks, written in his meticulous script with his ubiquitous Mont Blanc fountain pen, describe for instance detailed strategies for implementing the triple quadrupole in the late 1970s and electrospray ionization in the late ‘80s, as well as his long-term goal in the 1990s of moving his research toward immunology. In the earliest of the notebooks, he also outlines steps toward securing tenure: how many papers he would need to read per month and how many to write in support not only of his research but also his aspiration for tenure.

Don stayed abreast of the literature by implementing an extremely efficient approach to reading papers: First, he’d read the introduction and then the conclusion; only after absorbing the information in these sections would he decide whether it was worth his time to read the entire paper. To maximize his chances for success publishing research and obtaining grants, he taught himself how to write clearly and persuasively, referring often to the books on grammar and writing he kept on his desk. Perhaps most important in this process, he sought out what reviewers looked for in papers submitted for publication and what funding agencies wanted to see in grant applications.

The early 1970s were a heady time in Don’s lab. Virtually the same age as his graduate students, Don was easily able to convey his enthusiasm for the chemistry they were studying and to create both camaraderie and competition among the denizens of the lab. Always deeply connected to his family, Don left faithfully at 5:00 PM each evening to have dinner with his wife and children, only to return around 9:00 PM to work till well past midnight. The students, with no such 5 PM family commitment, worked straight through and were ready to leave about the time he returned, creating a certain amount of tension around the question of who was more dedicated to the work than whom.

The departmental mass spectrometers (MS) Don inherited upon arrival consisted of a Hitachi RMU-6e and an AEI MS-902. The latter had only an electron ionization source with a solids probe for entering samples. It had no gas chromatograph (GC) or liquid chromatograph (LC, the invention of functional LC/MS being more than a decade away). Measuring compounds having a molecular weight as high as 1000 was all but impossible. “High”-molecular-weight compounds volatile enough to be introduced via the solids probe were unlikely to produce a visible molecular ion; however, that was often of little consequence, because finding high mass peaks of intensity sufficient to allow counting on the light-beam oscillograph UV-sensitive paper trace that constituted the recording device output was unlikely. The possibility of multiply-charged ions was not even considered.

Intrigued by a technique gaining prominence in the literature, Don had immediately seen the potential of Chemical Ionization (CI) with novel “reagent gases” for the study of chemical reactions in the ion source of a mass spectrometer and made the purchase of a CI source for the MS-902 a part of his recruitment package at UVA. This innovative addition to the MS-9 had been developed for Hank Fales at the National Institutes of Health (NIH) by Marvin Vestal at Scientific Research Instruments Corp (SRIC) (3); however, with Marvin off to pursue a PhD at the University of Utah when it came time to install the source on the MS-902 at UVA, it fell to none other than Al Yergey, who had taken Marvin’s place at SRIC, to do the installation in Charlottesville.

Even in those early days, the mass spectrometer could also be interfaced with a Digital PDP8 computer. The software could be loaded via its paper tape reader to render the PDP8 capable of acquiring, calibrating, and printing mass spectra, although not of controlling the instrument. The PDP8 also had an external disk drive with a whopping 2.5 MB storage capacity (as best we recollect) for holding the collected data.

Unfortunately, the CI source, which had to be operated at an accelerating voltage of 8 kV to be compatible with the MS-902 electronics, was prone to arcing. These arcs invariably corrupted the computer operating program, which then had to be reloaded—in other words, the computer often seemed more trouble than it was worth. Once, however, when the computer failed and I (CNMcE) decided to fix it, it did teach me a valuable lesson. In those days you did not need glasses or a magnifying glass to see the components, and the wiring that connected them was wound around metal posts. Schematics were available and voltages could be read using a volt-ohm meter. Despite my best efforts, I failed to fix the computer that day. The very next day a capable service engineer came to the lab. On the second or third day of his visit, I asked him how the repair was going and he replied, “Oh, I fixed what was wrong in the first place in an hour. The rest of the time has been spent fixing the problems you caused by connecting a 120 V post with a 5 V post.” Although he fostered competition, Don never complained about our failures.

The CI source allowed chemistry in the gas phase to be studied. Don’s lab did the first gas phase deuterium exchange experiments to determine the number of active hydrogens on a molecule (4). In one study by James Ryan, nitric oxide (NO) was studied as a reagent gas (5). However, because of its oxidizing power, NO rapidly burned out the filament necessary for its own ionization, leading the Hunt group to develop a Townsend discharge for reagent gas ionization in the CI source (6), predating by a few months the report from Evan Horning’s lab at Baylor University of a corona discharge atmospheric pressure (AP) ionization source (7).

On a personal note, PhD in hand, I (CNMcE) left Don’s lab for DuPont Central Research Department in the early ‘70s. Some years later the Director of Analytical, after reading a paper from Don’s lab on derivatization to greatly enhance the sensitivity of negative ionization (see PJG), suggested that tetracyanoquinodimethane (TCNQ) might be a good negative ion derivatizing agent. It wasn’t, but under CI conditions TCNQ trapped radicals formed in the source in such a manner that allowed the chemical structure around the radical site to be determined using MS/MS fragmentation. This work earned me five JACS publications (8) and a promotion.

**Fig. 1:**
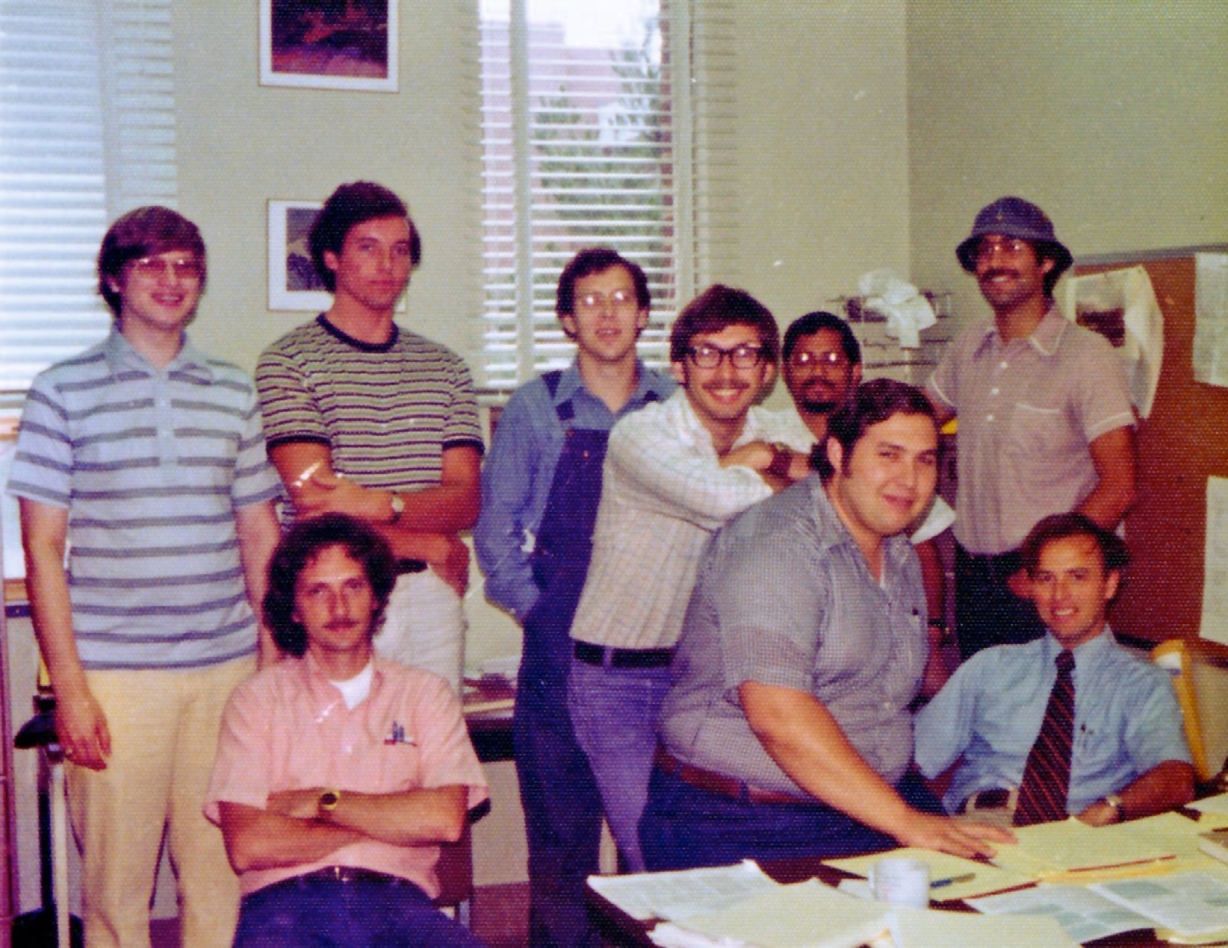
**Hunt Group in 1976.** L-R of *back row*: George Stafford, Alex Buko, Dave Lewis, Frank Crow, Satinder Sethi, Frank Botz; L-R of *front row*: Bill Brumley, Jeff Shabanowitz, and Don Hunt. How the composition of the group with respect to gender was about to change! Over the next 2 years, Exit George Stafford, Dave Lewis, Frank Botz, and Bill Brumley. Enter Anne Giordani, Mary Sisak, Jane Gale, Dottie Lane, and Mildred Coates.

Figure 1 shows denizens of the Hunt Lab in 1976. By the time I (PJG) joined the lab as a postdoc in late 1977, Don’s research group was really hopping–perhaps in part but not solely due to the change in the group's gender composition.

**Fig. 2:**
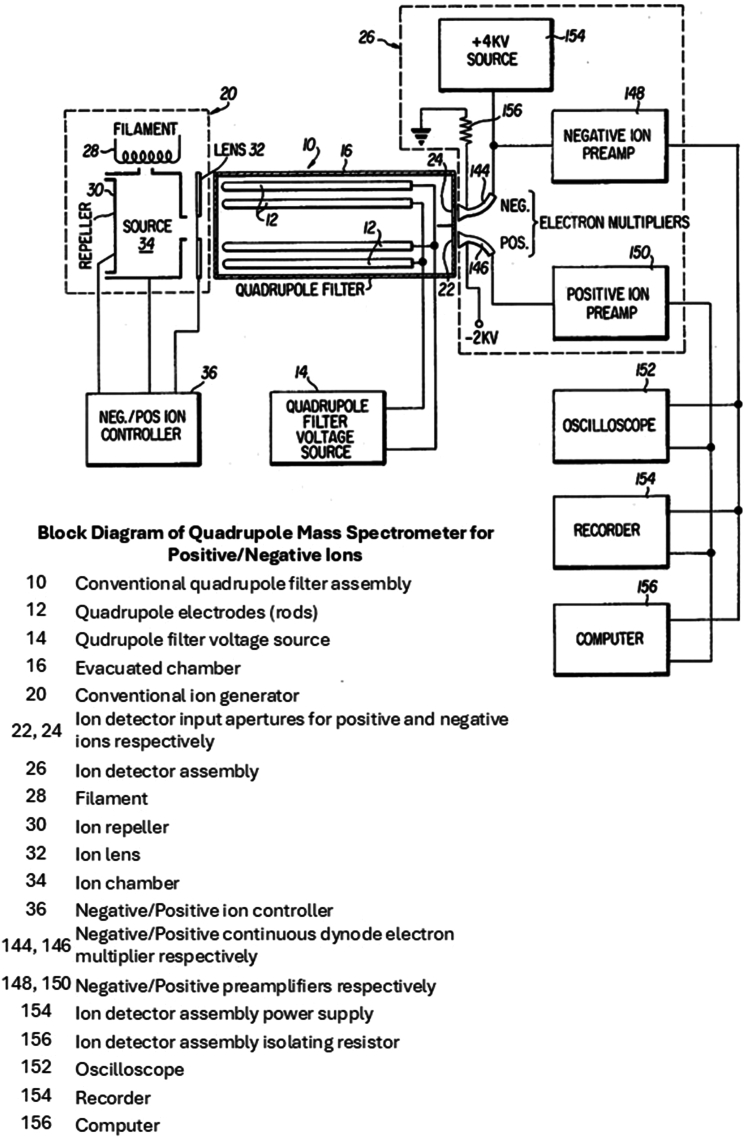
**Schematic of Pulsed Positive Negative Chemical Ionization (PPNICI) mass spectrometer.** Drawing from Stafford patent US4066894A.

**Fig. 3:**
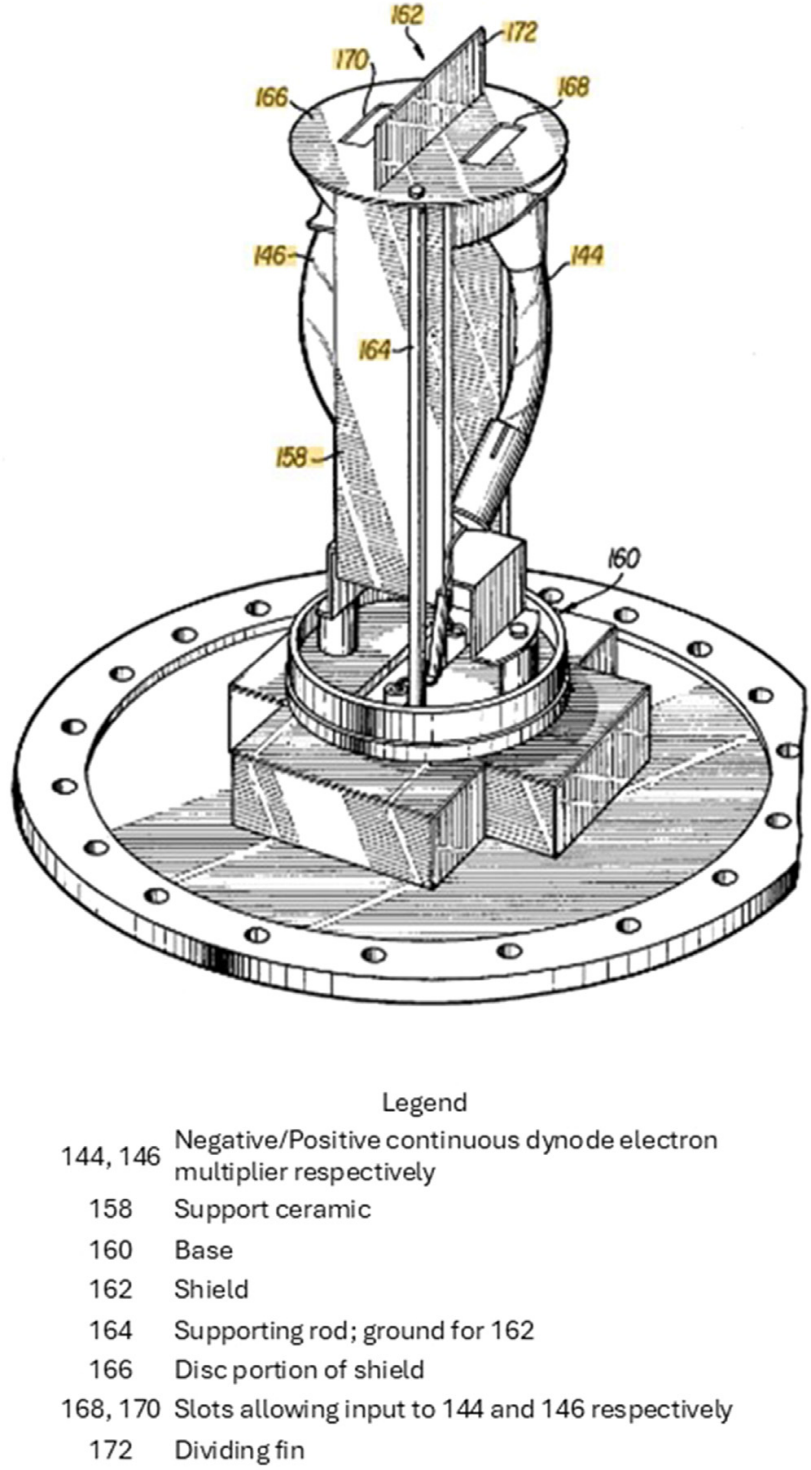
**Dual electron multiplier assembly.** Drawing from Stafford patent US4066894A.
When Don returned from his sabbatical, he came into the laboratory at UVA on his first evening home to get an update on his progress. I showed him the PPNICI instrument and some data I had obtained for aromatic compounds. I could tell he was not impressed when he indicated he did not see the value in the instrument. I had spent considerable effort designing and modifying the quadrupole mass spectrometer to be able to acquire PPNICI data and was a little discouraged, but I recognized Don was jetlagged from his flight home and thought I would talk to him again the next day. Indeed, the next morning when I talked to him, he was very excited about the PPNICI development, as he realized this would give us the capability to quickly and efficiently produce CI positive and negative ion spectra under the same ionization conditions, to greatly enhance compound identification using GC/MS. Figure 4 shows Don and me in his laboratory at UVA observing mass spectra generated from the PPNICI 3200 Finnigan QMS instrument.

Quadrupole instruments abounded thanks in part to the collaboration Don developed with Finnigan Corporation (later Thermo Electron and then Thermo Fisher Scientific), an alliance that would last for some 50 years [See Story, Syka, and Stafford, this volume.] The unique capabilities of these quadrupole instruments had already proved extremely useful: to wit, being able to produce data in both positive and negative CI modes. Intrigued by the complementary information that could be obtained by collecting both positive and negative CI spectra, in the mid-1970s graduate student George Stafford developed Pulsed Positive and Negative Ion Chemical Ionization (PPNICI) for essentially simultaneous collection of the spectra. As George remembers it,

I (GCS) started as a graduate student at the University of Virginia in 1973 and joined Professor Don Hunt’s research group the following year in 1974. At this time, GC/MS was already established as an excellent analytical technique for studying complex mixtures of molecules using electron impact ionization in the positive ion mode. Don wanted to explore the possible advantages of using positive and negative ion CI to improve compound detection and identification. There was a large selection of reagent gases that either had already been used or could be tried with CI, such as CH_4_, NH_3_, H_2_O, Ar, NO, O_2_, CH_3_ONO. The various reagent gases that could be introduced into the ion source would give the researcher a large selection of ionization energies and ion-molecule reactions to help with compound structure identification. Of particular interest, we wanted to see how the negative ion CI spectra would complement the positive ion spectra for analytical GC/MS applications.
A unique feature of the quadrupole mass filter is that it will transmit and analyze both positive and negative ions while operating under the same RF and DC scan voltage parameters and polarities. This meant you could develop a quadrupole mass spectrometer to analyze positive and negative ions simultaneously by rapidly switching ion source lens voltage polarities and developing a dual electron multiplier configuration capable of detecting both positive and negative ions. While Don was on sabbatical in England in the spring of 1975, I constructed the instrument shown schematically in Figures 2 and 3 from a Finnigan 3200 MS and a PerkinElmer GC, which I designed to be able to analyze and detect both positive and negative ions simultaneously in the same scan. We named this instrumental method Pulsed Positive Negative Ion Chemical Ionization (PPNICI) (9).

**Fig. 4:**
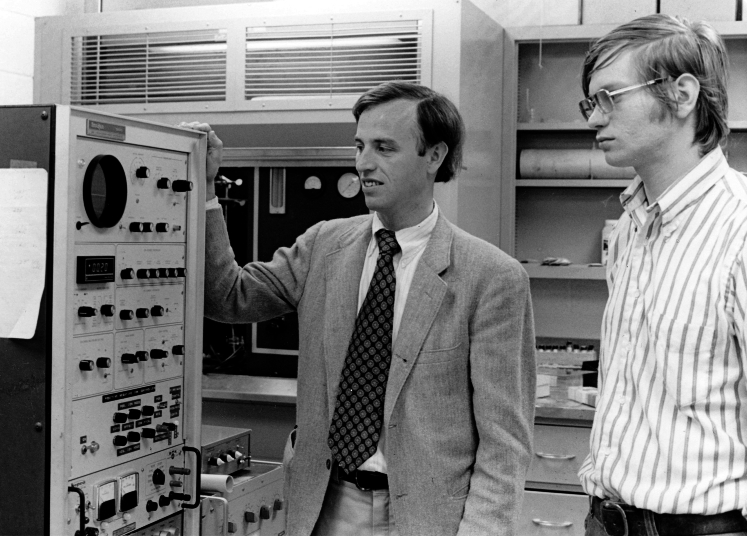
**Professor Don Hunt (*left*) and graduate student George Stafford (*right*) in the Hunt UVA laboratory operating the PPNICI 3200 Finnigan QMS instrument (circa 1976); PPNICI control module shown at the bottom in the instrument electronic console (*left*)**.
Don also suggested this would be a good analytical tool to measure the relative sensitivity response of compounds for positive and negative ion analysis. Using CI, we later investigated and determined that molecules with high electron capture affinities have 100 to 1000 times greater response in the negative ion mode compared to positive ion (10).

**Fig. 5:**
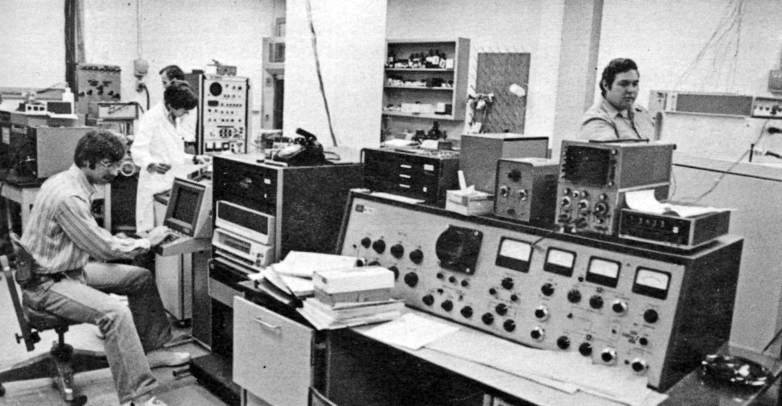
**Hunt Lab in the late 1970s.** L-R Frank Crow, Jane Gale, Don Hunt (behind PJG), and Jeff Shabanowitz.

Perhaps the most exciting development of the late 1970s in the Hunt Lab was Jeff Shabanowitz’s 1978 creation of the prototype of what would become the Triple Stage Quadrupole (TSQ) (11). Inspired by a presentation by Enke, Morrison, and Yost at ASMS that spring (12), Don prevailed upon Jeff to cobble together a prototype instrument similar to what they described from parts of three Hunt lab GC/MS systems — to the great frustration and consternation of the other graduate students, needless to say, given, as shown in Figure 5, the individual instruments' full utilization prior to the advent of the triple quad.

Don’s focus for all the instrumental innovations was their analytical utility for mixture analysis. Of particular interest was the identification of compounds categorized by the newly formed Environmental Protection Agency (EPA) as Priority Pollutants, and we students were charged with slogging away at component analysis in mixtures that could be as different as coal tar is from drinking water (13, 14). The prototype triple quadrupole in Jeff’s and Don’s hands demonstrated capabilities of such utility that its commercial counterparts would become the analytical instruments of choice for mixture analysis for the next decade and beyond (15, 16). However, commercialization of an instrument based on a prototype didn’t come easy.

While Bob Finnigan’s and Mike Story’s enthusiasm for the data Jeff was able to produce with the triple quad/collisional dissociation concept was great, Bob remained skeptical about the size of the market for such an instrument and challenged Don to find laboratories willing to *pre-*purchase the first five instruments Finnigan would produce. Despite his legendary personal shyness, Don was actually a great salesman and indeed was able to convince research groups at three industrial companies (including Phil Wadsworth at Shell Oil) and two universities to sign on, thus raising the ∼$2 million Finnigan would require to produce a commercial version of Jeff’s prototype [See Story *et al.*].

Don combined being a good salesman with being an excellent colleague and collaborator. At his retirement many years after that first triple quad purchase at Shell, Phil Wadsworth admitted that he had taken to heart a talk Don gave on using nitric oxide (NO) as a CI reagent gas to identify sulfur compounds. In Phil’s hands, NO was particularly useful for differentiating feedstocks in terms of their sulfur compound content (17). Indeed, the NO methodology proved so successful that it is still being used to identify total component structures and thiophenic sulfur in all types of crudes and feedstocks (18).

Another of Don’s industrial collaborations was with Phil Price at Union Carbide. Don first became interested in Phil’s work on CI energetics while Phil was still a graduate student at Minnesota. Later, after he moved to Union Carbide, Phil renewed the collaboration, visiting Don’s lab to see the CI source successfully installed on the MS-902 and to pick Don’s and Jeff’s brains about the tricks necessary to effectively interface CI sources to sector instruments. Don’s lab also collaborated on a project for one of Phil’s ‘internal customers,’ Union Carbide Carbon Products, whose carbon fibers were used for making, among other things, space shuttle cargo bay doors. To better characterize the extremely variable composition of the pitch from which the carbon fibers were made, Phil asked Don to explore the use of CI schemes (e.g., proton attachment and/or charge exchange) to better define the chemical composition of the pitch. This work kept several of us busy in the UVA lab for quite a while!

Don was always supportive of his students, even after they completed their degrees or postdoc appointments. To help solve a particularly knotty problem of compound identification in a plating bath dyestuff in the early 1980s, Don traveled to the RCA Materials Characterization Lab in Princeton NJ to work hands-on with UVA alum Bryan Bentz and me (PJG). Two days and many secondary ion mass spectrometry (SIMS) experiments later, we were able to show with some certainty that the “magic” compound contained in the dyestuff that accelerated the plating process was not the dye compound itself but a multimer of the dye’s basic structure. Later, Bryan and I, along with Charles Magee, another member of the RCA Materials Characterization Group as well as a UVA alum, were guests of Don and Jeff in the Hunt lab at UVA, where we used SIMS coupled with MS/MS to study polyatomic species present in a mixed oxide cathode and an NBS standard of Pb-Ba glass. As Charles says in an abstract of the paper he presented on this work at the 30th Annual Conference on Mass Spectrometry and Allied Topics in 1982, “The ability to obtain parent, daughter and neutral loss spectra from the collisional dissociation of selected parent ions produced by sputtering solid targets is [was] a significant addition to the characterization of inorganic solids by SIMS.” (19)

Despite all this success—or perhaps because of it—Don’s research interests in the late 1970s and early 1980s were drawn to the nascent field of what would come to be called proteomics (20). Back at UVA after a sabbatical in Dudley Williams’ lab at Cambridge in 1975, Don was determined to explore ways mass spectrometry could enhance the study of these biological molecules. His early work with Field Desorption emitters (21) showed promise in volatilizing without decomposition of ionic, highly polar, and thermally labile organic molecules (22).

Although his first thought had been to focus on oligonucleotides given the work he had done in Biemann’s lab on dinucleotides (2), Don was dissuaded by Williams and then post-doc Howard Morris (HRM), who pointed out that mass spectrometry would have a hard time competing with the then dominant Sanger Sequencing technology (23). Instead, Don and his students turned their attention to peptides.

**Fig. 6:**
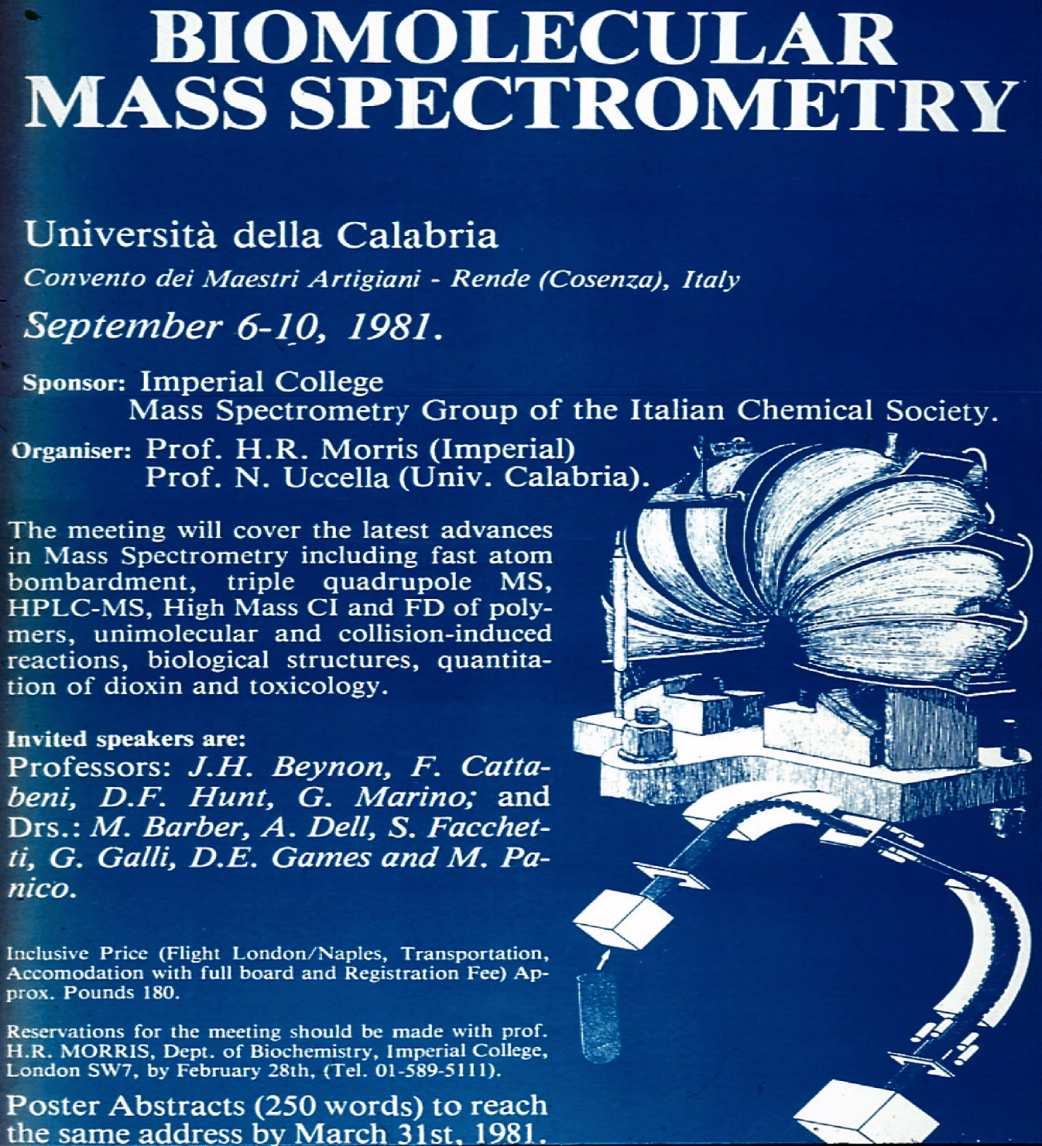
**Invitation to the Biomolecular Mass Spectrometry meeting in Calabria Italy in September 1981**.

Thanks in part to an extended sabbatical visit from Morris at Don’s invitation in 1978, by 1979 Hunt Group students were using CI of permethylated and acetylated peptides in MS/MS experiments to determine the structures of these building blocks of proteins. As Howard remembers those exciting days:

I first met Donald in the early ‘70s having been invited to do a post-doc (’70–72) with Dudley Williams at the University Chemical Laboratory in Cambridge (England), when Don called by to discuss his research on Chemical Ionisation. His visit happened to coincide with my giving a Group Seminar on advances in the mixture analysis strategy for MS protein sequencing which seemed to grab Don’s imagination, and after the seminar, we entered into intense discussions over a pint or two at the local pub in Lensfield Road. There was a common interest in the fundamentals of instrument design, in fragmentation mechanisms, and in micro-scale chemical modifications needed at that time to get involatiles into the vapor phase for mass spectrometry.
We met again in 1972 on my first visit to America at the ASMS Conference held in Dallas TX, where I presented on our “Mixture Analysis” strategy for *de novo* protein sequencing (24). In a moment of serendipity, I bumped into Don among the 1500 or so scientists at the meeting. His enthusiasm for his subject was both palpable and memorable. I say serendipity because Don and I subsequently formed a friendship that would last over 5 decades based on respect and the healthy competition of differing approaches to scientific solutions to important biological problems.
In 1977, Don invited me to take a 6-month Visiting Professorship at the University of Virginia starting in 1978, with both undergraduate and postgraduate teaching duties as well as research opportunities. There I witnessed the magic of Jeff Shabanowitz’s innovative instrumentation enhancements. To help introduce my methodologies for biomolecule analysis to his laboratory, Don assigned a very bright student, Alex Buko, to learn some of our protein MS protocols. It was a delight during those 6 months to work with such talented individuals.
As the 1980s dawned, it was clear to all that the next big advance in biopolymer analysis would need to be at the ionisation level. Field Desorption had led the way, and I had by then commissioned the building of a high field magnet for the next generation MS-902 called the Kratos MS50. This instrument had the mass range (>3200) on high-field magnet sector mass spectrometers needed to study the proteolytic digest products generated by enzymes such as Trypsin. Californium Ionisation had shown promise (25), and in-beam electron impact had demonstrated that direct analysis of involatiles might not be a pipe-dream at some point. It might even give equivalent data to a new simple design of Field Desorption source I had built in Cambridge in 1971 that allowed the correction of the Prelog antibiotic structure of Echinomycin by demonstrating an unequivocal molecular weight of 1100, which supported a Thioacetal rather than a Dithiane cross-linked structure (26).
With all this interest in biopolymers among mass spectrometrists, in 1980, I organized a “Soft Ionisation Mass Spectrometry” meeting at Imperial College and invited a number of international speakers, including Don, to describe their latest research. The meeting was a resounding success, not least because Michael Barber, who had just invented Fast Atom Bombardment (FAB) ionization (27), gave the first announcement of his results on that occasion.
The next year I invited Don to be a Visiting Professor in the Biochemistry Department at Imperial College and soon after a speaker at the 1981 Anglo-Italian Mass Spectrometry meeting in a delightful location in a hill-top Convent in Calabria, Italy. Nicola Uccella, the co-organiser of the event, had met Don nearly a decade earlier on one of his visits to Dudley Williams’s group where at the time Nicola was a PhD student working on the fundamentals of ion structure and metastable events. The invitation to the meeting in Calabria is shown in Figure 6.

With the advent of FAB, Don’s lab was able to demonstrate nmol sensitivity for these peptide fragments of biological molecules (28), and the next phase of his research was off and running. The direct analysis of higher molecular weight species that FAB made possible, however, only served to underscore the need for higher mass range and increased resolution. Leveraging the strong relationship he had developed with Finnigan a decade earlier, Don and his group would also continue in the next decade to be at the forefront of instrument development. [Story *et al.* chapter, Yates chapter]

Reflecting on our productive interactions and cherished friendships with Don, we four note the following:

For me (CNMcE), joining Don’s group was a real blessing. He was actually fun to work with, taught me a great deal, helped me get exceptional jobs, and remained a friend over the years. I certainly owe Don a debt of gratitude!
In my early graduate studies with Don, I (GCS) was always impressed by the excitement he would bring and openly express for the exploration of new scientific methodologies.
Don’s love for developing new ideas and pushing science forward always inspired me and my fellow graduate students to do our best work each day and to stay focused on achieving our objectives.
This tribute to Donald follows my (HRM) first when he celebrated his 65th Birthday in 2006 (29), and it gives me great pleasure to make it. It has also been a great pleasure for Maria and me to support the Donald Hunt Graduate and Postdoctoral Research Fund at the University of Virginia and the excellent work they are doing in transferring Don’s knowledge, work ethic, and values onto a new generation of chemists. I am privileged and honored to pay tribute to Donald’s seminal contributions to the field of science, and Maria and I wish him a long, happy, and well-deserved retirement!
Don’s influence on the beginning of my (PJG) career was profound. Trained as a physical chemist, I was both delighted and challenged to find in Don’s lab ways to use ion-molecule reactions—my first love—for practical, that is, analytical, purposes. Don’s support has endured throughout both my professional and personal life. His willingness to share his wisdom, his guidance, and even a deep connection with his family, has been unstinting. Like many others, I have been truly fortunate to have had Don not only as my mentor but also as my friend.

## Conflict of interest

The authors declare that they have no conflicts of interest with the contents of this article.
